# Sepsis-associated myocardial injury: Incidence and mortality

**DOI:** 10.1097/MD.0000000000042513

**Published:** 2025-06-20

**Authors:** Ye-Ting Zhou, Guang-Sheng Wang, Xin-Chun Gao, Shao-Dan Wang, Si-Wei Wang, Dao-Ming Tong

**Affiliations:** a Affiliated Shuyang Hospital, Xuzhou Medical University, Jiangsu, China; b NICU, Affiliated Shuyang Hospital, Xuzhou Medical University, Jiangsu, China; c CCU, Affiliated Shuyang Hospital, Xuzhou Medical University, Jiangsu, China; d Department of Intensive Care Medicine, Affiliated Shuyang Hospital, Xuzhou Medical University, Jiansu, China; e Emergency Department, Affiliated Shuyang Hospital, Xuzhou Medical University, Jiangsu, China; f Medical Research Department, Affiliated Shuyang Hospital, Xuzhou Medical University, Jiangsu, China.

**Keywords:** myocardial injury, myocardial injury markers, outcome, risk factors, sepsis

## Abstract

The incidence and mortality associated with sepsis myocardial injury (SAMI) remain understudied. We aimed to explore whether the 4 myocardial injury markers (MIMs) scores in the Intensive Care Units (ICU) were associated with the incidence and mortality of patients with SAMI. A retrospective study collected data from 316 adult SAMI patients who underwent MIMs tests on the ICU (January 1, 2017–January 1, 2020) of the Shuyang Hospital in China, and the predictors of mortality were determined using multivariable Cox models. Between January 1, 2017 and January 1, 2020, 316 (61.1 %) adults were diagnosed with SAMI in a consecutive sample of 517 admissions. A total of 177 (56.0%) patients with SAMI died at 28 days follow-up, and its initial (24 hours) MIMs score, highest MIMs scores (>80 hours), sepsis-associated organ failure assessment (SOFA) score, systemic inflammatory response syndrome (SIIRS) point, and inflammation markers were significantly different between the survival and non-survival group (all *P* < .05). We found that a initial elevated MIMs score (hazard ratios [HR], 6.4; 95% CI = 4.298–11.48), high SIRS point (HR, 3.2; 95% CI = 1.249–5.115), high SOFA score (HR, 3.6; 95% CI = 1.315–5.974), and highest MIMs score (HR, 6.8; 95% CI = 4.379–11.53) were associated with high mortality for SAMI. The area under the ROC curve for mortality of SAMI was significantly larger for the highest MIMs score (0.88, 95% CI = 0.85–0.96) than for the initial MIMs score (0.84, 95% CI = 0.80–0.87) (*P* < .001). High MIMs scores in SAMI was associated with high mortality, suggesting that a greater need to predict outcomes and active treatment SAMI to reduce mortality, in addition to timely antibiotic treatment.

## 1. Introduction

According to our opinion, cardiovascular dysfunction with elevated myocardial injury markers (MIMs) can be roughly divided into 2 types: myocardial infarction associated cardiovascular dysfunction (MACD)^[1,2]^ and sepsis associated myocardial dysfunction (SACD).^[3,4]^ Elevated levels of MIMs is a common condition following MACD and/or SACD in the intensive care units (ICU).^[5,6]^ Previous research has shown that MACD was associated with short-and long-term mortality, which was related to the the Intermountain Risk Score and involved in 23.1% mortality.^[1,2]^ In global, sepsis is a high mortality disease, even so high as to > 70% mortality.^[7,8]^ Although sepsis associated myocardial injury (SAMI) as a serious consequence of sepsis, evaluation of MIMs score to predict the mortality and risk of death remain understudied. We hypothesized that the critical ill adults due to infection would be having a high prevalent SAMI and with very high mortality, which may be predicted by elevated 4 MIMs scores. The purpose of this study is to determine whether the incidence and mortality of SAMI is involved elevated 4 MIMs score. We hope that our study results will verity this hypothesis and provide evidence of predicting poor outcome for SAMI in the ICU, so that to improve early diagnosis and management.

## 2. Methods

### 2.1. Patients source

In this retrospective observational study, we conducted a consecutive sample of the critical ill adult patients, and they were from the emergency department by related test diagnosed as sepsis-associated SACD and sent into ICUs, including a general ICU, a NICU, and a CCU in the Affiliated Shuyang Hospital of Xuzhou Medical University (between January 1, 2017 and January 1, 2020) with a length of stay more than 24 hours. The study is full compliance with the Helsinki declaration, and approved by the ethical committee on clinical research of the Affiliated Shuyang Hospital of Xuzhou Medical University. Due to this study only involved a review of records obtained as a part of routine medical care, it did not require written information consent from all patients. All the author ensure for the accuracy and completeness of the data/ analyses and for the fidelity of this report to the study, which is available with the full text of this article.

### 2.2. Procedures

In the initial ICU admission, we tested the levels of 4 MIMs [troponin I, myohemoglobin, creatine kinase isoenzyme MB (CK-MB), and α-hydroxybutyrate dehydrogenase (HBDH)]. According to the records of 4 MIMs measured in the ICUs, also refer to previous studies,^[8–10]^ in this study, a sepsis-associated myocardial injury (SAMI) diagnostic criteria was as follow: Infection must occur first, followed by the presence of the following 3 conditions: with evidence of 1 or more organ dysfunction; also with the elevated troponin I (required) and any or more of the following 3 additional items: elevated myoglobin; elevated creatine kinase isoenzyme MB (CK-MB) and elevated α-hydroxybutyrate dehydrogenase (HBDH).

We excluded those critical ill patients: patients of either death or automatically out of the ICU due to family asked to give up treatment within the first 24 hours; those with elevated MIMs from septic shock or non-sepsis diseases such as the ST elevation myocardial Infarction; and those heart diseases involved elevated troponin I, such as myocarditis/pericarditis, etc.

We also used the SAMI score to assess its severity: isolated elevated troponin I indicates SAMI score = 1; any troponin I increase + additional item indicates SAMI score = 2; any 2 troponin I increase + additional items indicate SAMI score = 3; Troponin I increase + all other items indicate SAMI score = 4.

We retrospectively collected the data from electronic medical records in the ICUs, which was entered directly into our hospital database. The patients who was meet the diagnosis of the elevated MIMs due to infection were analyzed in this study.

According to sepsis-3 criteria,^[9]^ in this study, the diagnosis of sepsis was cleared by emergency CT screening and sepsis-associated organ failure assessment (SOFA). The diagnosis of infection includes suspected infection (SIRS > 2 points) and confirmed infection.

The confirmed infection main indicate the community-acquired pneumonia (CAP). The CAP is characterized by an airspace consolidation in 1 segment or lobe, particularly the lower lobes. We calculated the SOFA score from the initial day to 15 days in the ICU. We also collected the laboratory other findings, included the elevated interleukin-6 (IL-6), C-reactive protein, PaO2/Fio2 ratio, APACHE II score, electrocardiogram (ECG), and echo-cardiography and etc.

### 2.3. Primary outcomes

The primary outcomes of the study was the composite outcome of in-ICU incidence and mortality for SAMI patients. Mortality rate included at initial 4 days and the 28 days follow-up in the ICU. If the patient gave up treatment in the ICUs, in order to assessment the mortality of patients, survival state was confirmed by a main study coordinator who examined the patients’medical records and at the 28 days follow-up.

### 2.4. Statistical methods

Based on the inclusion criteria of SAMI, the study data were divided into the SAMI survivor group and non-survivor group for statistical analysis. The following variables should be included in the 2 groups analysis: MIMs scores in the first day aggregated as the median from 4 MIMs (troponin I, CK-MB, HBDH, and myohemoglobin); laboratory tests in the first day aggregated as the median from a maximum of assessments (IL-6, CPR, D-dimer, procalcitonin, SIRS points, GCS scores, PaO2/Fio2 ratio, and creatinine levels); and SOFA scores were not included septic shock score. To evaluate risk factors associated with the poor outcomes, we must be used a Cox regression model with hazard ratios (HR) and 95% confidence intervals when they were significant in the univariate analysis. The subgroups analysis from within the initial 1 day to 15 days after ICU admission in figures aggregated as the median from assessments, including IL-6, CPR, D-dimer, procalcitonin, SIRS points, and etc, which used the Mann–Whitney *U* test or nonparametric test. To assess the correlation of outcomes between the continuous variables should be used a Pearson correlation coefficients, which were to explore the relationships between elevated MIM score as well as SOFA score and mortality. The cumulative incidence of SAMI was showed in figures. The 28-day survival was plotted in a curve and analyzed. In addition, comparisons of the areas under the receiver operating characteristic (ROC) curves were also performed.^[10]^ The results for all patients are expressed as the mean ± standard deviations or median (IQR) for continuous variables, and n (%) indicates qualitative values. All statistical analyses were performed with the use of SAS software, version 9.3, and R software, version 2.13.2.

## 3. Results

**Figure 1.**
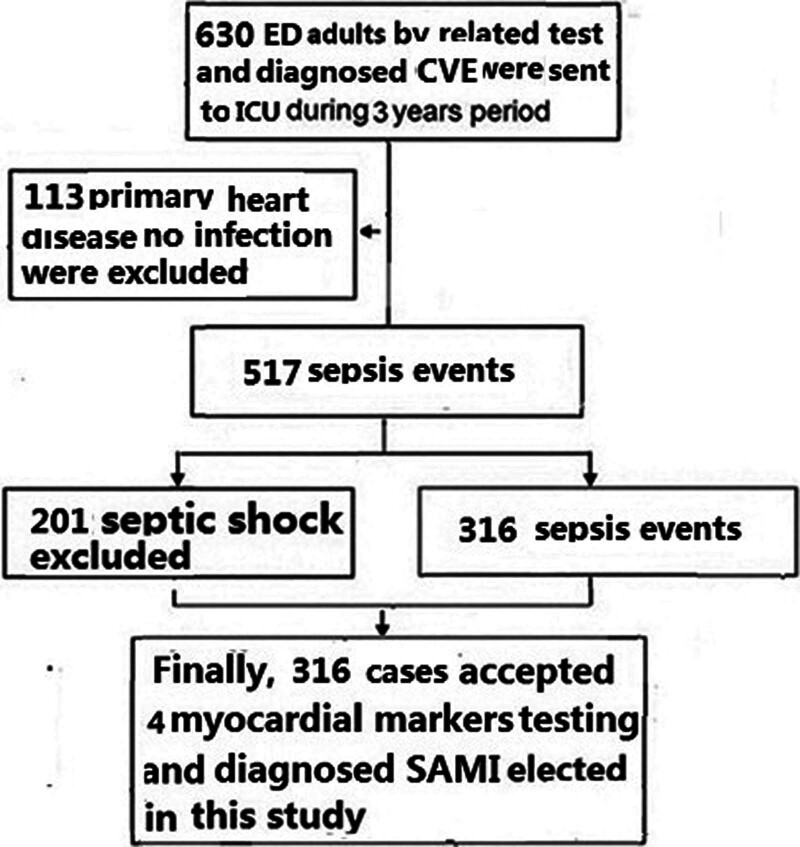
Flow chart. The patients who were not met the eligibility for this study design were excluded. Finally, 316 SAMI were included in the present study. SAMI = sepsis-associated myocardial injury.

From Jen 2021 through Des 2023, A total of 630 acute critically ill adult patients with SACD data in consecutive sample of ICUs were recruited. After ruling out patients who did not meet the eligibility criteria for this study design, 316 (60.1%, 316/517) SAMI were diagnosed and analyzed in this study population (see Fig. 1).

**Figure 2.**
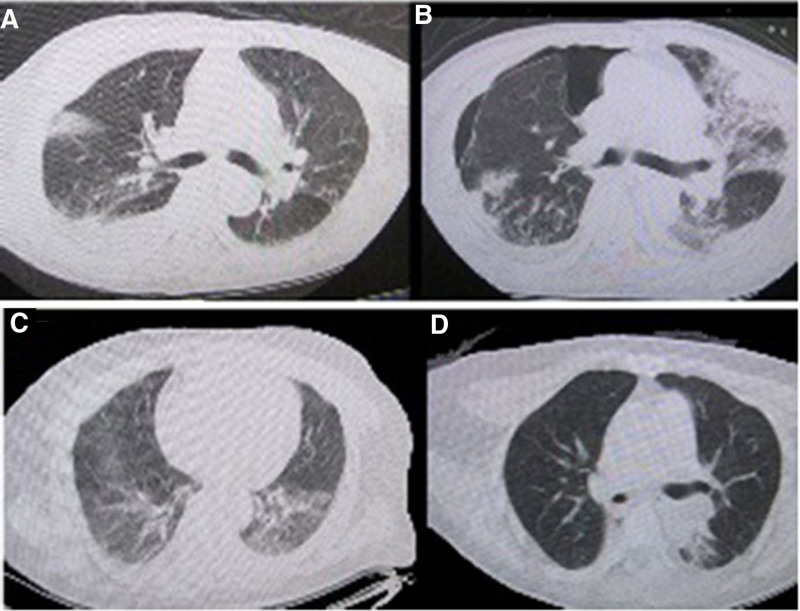
(A and B) is from a patient with cough and dyspnea for 2 days, chest CT on ICU demonstrated that the opacity in the bilateral lungs (A) and Lab tests showed elevated 4 MIM and patient was in respiratory failure requiring mechanical ventilation. on ICU day 16, CT showed pneumonia (B) had not improved, and the patient was diagnosed with SAMI following CAP and died in the ICU. (C and D) is from a patient with bilateral pneumonia on the day after onset, chest CT demonstrated that the bilateral pneumonia (C) Lab tests confirmed elevated 4 MIM. On ICU day 3, patient was in respiratory failure requiring mechanical ventilation and The patient was diagnosed with SAMI. On day 6 days, chest CT showed pneumonia have improved (D) and finally recovery. CAP = community-acquired pneumonia, ICU = intensive care units.

**Figure 3.**
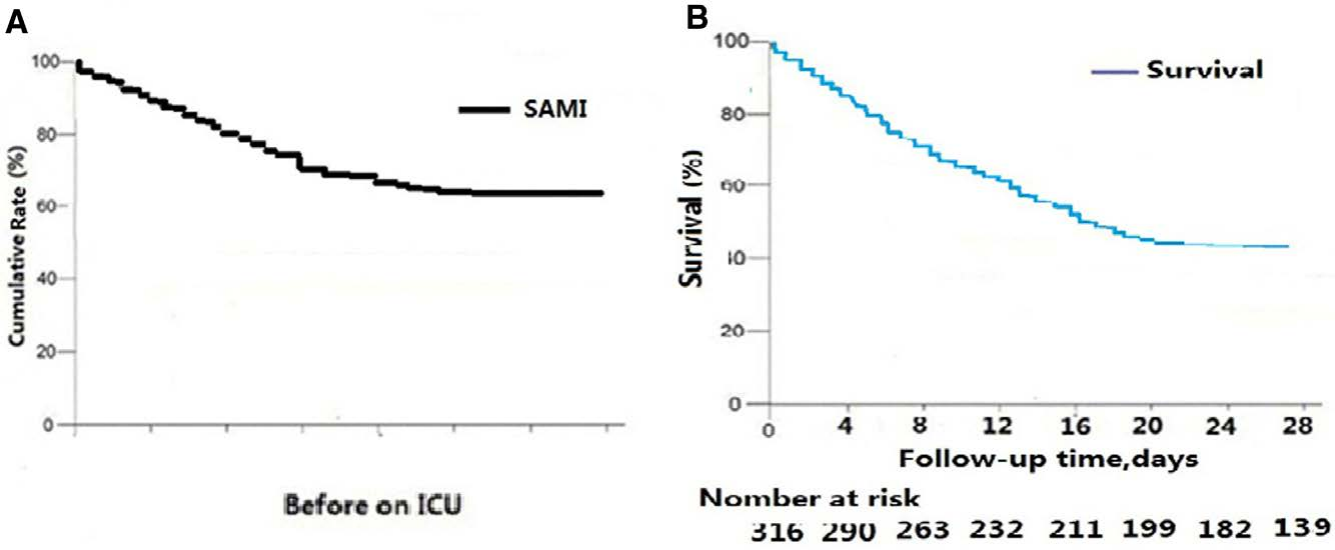
The incidence rate before on ICU and the survival rate of SAMI at 28 days follow-up in the ICU.

In the present study, the clinical characteristics of 316 SAMI patients are shown in Table 1. Among 316 SAMI patients, 201 (64.4%) patients were males, with a mean age of 67.8 ± 16.1 years. The median time from symptoms onset to ICU admission was 3.2 hours (range, 0.5–240). The initial assessment of MIMs showed that the most frequent SAMI was MIMs of 4 (70.1%), followed by MIMs of 3 (20.0%) and MIMs of 2 (9.6%), but isolated elevated MIM was rare (0.4%). The most common initial presenting myocardial injury symptom was acute dyspnea (306 [98.1%]) with 257 (94.1%) need invasive mechanical ventilated on the ICU admission, followed by tachycardia/ arrhythmia (273 [87.5%]). All patients had normal or increased blood pressure rather than hypotension. Some patients experienced chest pain (12 [0.04%]), even sudden cardio arrest (8 [0.026%]) with received cardiopulmonary resuscitation (CPR). The initial infection was CAP (81.1%) (see 2 cases in Fig. 2). Cumulative Rates of SAMI prevalence before on ICU and survival rate during 28 days in the ICU for ASMI population is showed in Figure 3A and B.

**Table 1:** Clinical characteristics in patients with SAMI (n = 316).

Characteristic	Value
Age (yr, mean ± SD)	66.8 ± 16.1
Male gender (n, %)	203 (64.2)
Median (IQR) time from onset to ICU (h)	3.2 (0.5–240)
Comorbidities (n, %)	
Hypertension	145 (45.8)
Cardiac-cerebral vascular disease	108 (34.3)
Diabetes	52 (16.5)
Chronic lung disease	46 (14.4)
Cancer	28 (0.9)
Initial presenting symptoms (n, %)	
Dyspnea	310 (98.1)
Tachycardia/arrhythmia	277 (87.5)
Fever	176 (55.8)
Altered mental status	240 (76.0)
Chest/abdominal pain	18 (5.6)
Dizziness	16 (5.2)
Other	7 (2.3)
Imaging findings by CT scan on ICU (n, %)	
Community-acquired pneumonia	259 (82.1)
Abdomen infection	43 (13.5)
Ureteral obstruction with infection	8 (2.6)
Other infection	6 (1.9)
Later positive bacteria culture in sputum	282 (89.2)
Later positive bacteria culture in blood	96 (30.3)
Measured myocardial injury markers on ICU (n, %)	
Elevated 4 MIMs	222 (70.1)
Elevated 3 MIMs	63 (19.9)
Elevated 2 MIMs	30 (9.6)
Elevated 1 MIM	1 (0.4)
Median (IQR) initial GCS score	13 (5–15)
Median (IQR) temperature (°C)	37.5 (35.3–40.0)
Median (IQR) arterial pressure (mm Hg)	107 (70–168)
Median (IQR) heart rate (beats/min)	106 (98–137)
Median (IQR) respiratory rate (breaths/min)	27 (24–45)
Intubation and IMV	298 (94.2)
Antibiotics treatment within initial 3 h on ICU, n (%)	316 (100.0)
LOS (d, IQR)	9 (1–56)
Mortality at 15 days, n (%)	142 (45.0)
Mortality at 28 d follow-up, n (%)	177 (56.0)

Abbreviations: GCS = Glasgow coma scale, ICU = intensive care unit, IMV = invasive mechanical ventilation, IQR = interquartile rang, LOS = length of stay, SAMI = sepsis-associated myocardial injury, SAMIM = sepsis-associated myocardial injury marker.

**Figure 4.**
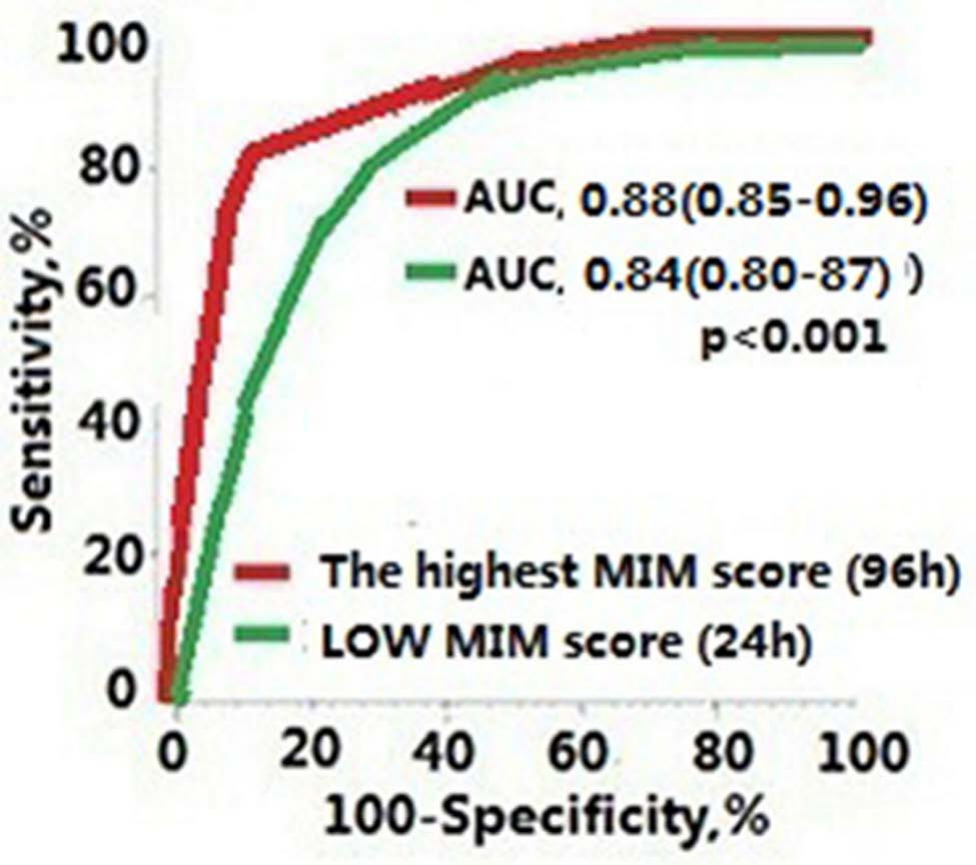
The area under the ROC curve was significantly larger for the highest MIMs score than for the initial MIMs score (*P* < .001). ROC = receiver operating characteristic.

The highest MIMs score presented the largest area under the ROC curve (0.88, 95% CI = 0.85–0.96) when the cutoff value was 3.8 scores (median), the sensitivity was 87.5%, and the specificity was 99.6%. The area under the ROC curve was significantly larger for the highest MIMs score than for the initial low MIMs score (area under ROC curve 0.84, 95% CI = 0.80–0.87) (Fig. 4, *P* < .001).

### 3.1. Univariate analysis and Cox regression of SAMI

The univariate analysis of the non-survival and survival SAMI patients are described in Table 2.

**Table 2:** Baseline characteristics in patients with SAMI who non-survivor and survivor in ICU admission (n = 316).

Variables	Normal value	Non-survivor (n = 177) Median (IQR)	Survivor (N = 139) Median (IQ)	*P*-value
Age (yr)	>18	65 (52–91)	63 (48–89)	302
Initial MIMs scores (24 h)	0	3.6 (2–4)	2.3 (1–4)	.000
Troponin I (ng/mL)	0 to 0.04	5.9 (2.6–50.3)	2.3 (0.13–28.7)	.000
Myohemoglobin (µg/L)	21 to 110	537 (125–1500)	213 (112–358)	.000
CK-BM (U/L)	0 to 25	52 (29–364)	29 (26–356)	.000
HBDH (U/L)	72 to 182	353 (183–1478)	183 (87–779)	.000
The highest MIMs scores (>80 h)	0	3.8 (3–4)	3.1 (1–4)	.000
Lower MIMs scores (15 d)	0	2.3 (1–3)	1.9 (1–3)	.000
White blood cell count, ×10^9^/L	3.5 to 9.5	13.0 (8.7–34.6)	12.3 (2.3–25.7)	.417
Neutrophil percent, %	40 to 75	84.7 (66.6–97.2)	85.7 (56–96.4)	.599
Platelet count, ×10^9^/L	125 to 350	177 (23–887)	168(3.0–493)	.265
Interleukin-6, pg/mL	<7	24.6 (9.6–4000)	11.2 (3.6–91)	.000
C-reactive protein, mg/L	0 to 8	122 (4.2–379)	102 (4.2–378)	.036
Procalcitonin, ng/mL	0 to 0.05	2.7 (0.03–500)	1.2 (0.3–35.4)	.034
D-dimer, mg/mL	0 to 0.232	1.9 (0.07–94.7)	1.2 (0.17–67.8)	.040
Lactic acid, mmol/L	0.7 to 2.1	3.2 (1.2–12.2)	2.8 (1.2–15.9)	.339
Serum glucose, mmol/L	3.9 to 6.1	8.6 (3.6–44.5)	7.9 (4.0–41.0)	.210
Creatinine, µmol/L	64 to 104	99.6 (38.99–739)	72 (10.4–494)	.019
Total bilirubin, mmol/L	0 to 23	29.2 (6.2–1245)	19.1 (4.7–181)	.043
SIRS ≥ 2	<2	3.4 (2–4)	2.1 (1–4)	.000
GCS score	0 to 15	6.1 (9–11.8)	10.3 (9–12.2)	.021
PaO_2_/FiO_2_, mm Hg	>400	106 (80–210)	186 (180–370)	.013
SOFA score	0	8.3 (3–11)	5.1 (3–11)	.000
APACHE II score	<15	19.5 (15–45.5)	17.5 (5.5–38.5)	.031
LOS in ICU (d)	>1 to 28	7.1 (1–28)	15.0 (2–56)	.002

APACHE II = acute physiology and chronic health evaluation, CK-BM = creatine kinase isoenzyme MB, GCS = Glasgow coma scale, HBDH = α-hydroxybutyrate dehydrogenase, LOS = length of stay, MIM = myocardial injury marker, SAMI = sepsis-associated myocardial injury, SIRS = systemic inflammatory response syndrome, SOFA = sepsis-associated organ failure assessment.

Trends in the MIMs scores during the ICU stay were analyzed. The univariable analysis showed that a numerical decrease in the initial MIMs score was associated with a mortality rate <50.0%. While a numerical increase the highest MIMs score was associated with a mortality rate >50.0% (Table 2).

During the ICU stay, these elevated infection markers over time has not significant decreased (all *P* < .05; Fig. S1, Supplemental Digital Content, https://links.lww.com/MD/P47). while the MIMs scores, PaO2/Fio2 ratio, SIRS points, SOFA scores over time has not significant improved (all *P* < .005; see Fig. S2, Supplemental Digital Content, https://links.lww.com/MD/P47). Moreover, the GCS scores, creatinine levels, total bilirubin levels, APACHII scores, and the length of stay were also significantly different between the 2 groups. However, by Cox regression analysis, an initial elevated MIMs scores (HR, 6.4; 95% CI = 4.298–11.48), a high SIRS points (HR, 3.2; 95% CI = 1.249–5.115), a high SOFA scores (HR, 3.6; 95% CI = 1.315–5.974), and a later highest MIMs scores (HR, 6.8; 95% CI = 4.379–11.53) were established as death risk factors of non-survivors (Table 3). All patients had the abnormal ST-T changes in ECG, and more than 90% patients who were underwent an echo-cardiography had the ejection fraction <50%. The subsequent data confirmed that most patients had a positive sputum, but positive blood culture were 29.0% of SAMI (see Table S1, Supplemental Digital Content, https://links.lww.com/MD/P47).

**Table 3:** The Cox regression analysis in septic patients with high mortality in ICU.

Variables	HR	95% CI for HR	*P*-value
Initial elevated MIMs scores	6.1	4.298 to 11.48	<.0001
SIRS points	3.2	1.249 to 5.115	<.0001
SOFA scores	3.6	1.315 to 5.974	<.0001
Highest MIMs score	6.8	4.379 to11.53	<.0001

CI = confidence interval, HR = hazard ratio, MIMs = myocardial injury markers, SIRS = systemic inflammatory response syndrome, SOFA = sepsis-associated organ failure assessment.

### 3.2. Outcomes and related assessment

During the initial 15 days of the ICU, the mortality of SAMI patients was 45% (142/316). The fatality of SAMI was 56.0% (177/316) at 28 days follow-up. In this study, we also assessed the SAMI related subsequent multiple organ failure (MOF) at the 28 days in the ICU (Table S2, Supplemental Digital Content, https://links.lww.com/MD/P47), which also involved the poor outcomes. The correlation coefficient for mortality showed that highest (>80 hours) MIMs score (*R* = 0.75, 95% CI = 0.6609–0.8226) was higher than initial MIMs score (*R* = 0.70, 95% CI = 0.5764–0.7729) and SOFA score (*R* = 0.68, 95% CI = 0.5959–0.7846) at 28 days in ICU (Fig. S3, Supplemental Digital Content, https://links.lww.com/MD/P47).

## 4. Discussion

In this population study, we found that the prevalence of SAMI was in 60.1% on the ICU admission, which indicates that SAMI was a high prevalence of acute cardiovascular dysfunction and was with 57.5 % of high mortality at 28 days follow-up. The median age of patients was 66.8 years. Male sex was in 64.2% (203/316) of the patients. Chronic hypertension (45.8%), cardio-cerebral vascular comorbidities (34.3%), diabetes (16.5%) and chronic lung diseases (14.4%) were frequent among most patients. In fact, The chronic complications have been reported to influence individual outcomes.^[11,12]^

Our study found that the clinical features of these SAMI patients was mainly associated with elevated MIMs and myocardial injure due to infection. To our best knowledge, SAMI with elevated MIMs due to infection is still understudied. Moreover there was more reported on elevated α-hydroxybutyrate dehydrogenase as a relationship with SAMI in recent studies.^[13,14]^ The myocardial injure involved multiple MIMs release from the injured heart into the circulation.^[5,6,13,14]^ This is a result of systemic inflammatory response which can lead to coagulation dysfunction, ischemic injury/ cell death, and acute organ failure.^[15–17]^

In our study, the initial elevated MIMs scores in SAMI were presented as reliable predictor of incidence before ICU admission. The highest MIMs scores (>80 h) indicated the degree of SAMI over time and could be a useful tool in clinical to predict the outcome of patients. Knowing these 2 scores, we should promote active early treatment to reduce mortality. The equivalence of the area under the ROC curve for both parameters suggest that they are equally effective in predicting the outcome. And, the reliability of the both elevated MIMs scores to predicting mortality rate was supported by Cox regression and coefficient analyses

The high mortality of SAMI can be explained in 2 respects. One respect, the evidence from current data confirmed that the CAP was the leading cause of infection. Acute pneumonia involved in the systemic inflammation resulting in a dysregulated immune response and leading to endothelial injury and/or capillary leak syndrome.^[17,18]^ Furthermore, capillary leak syndrome is also due to an overwhelming release of pro-inflammatory cytokines as well as anti-inflammatory factors.^[19]^ The inflammatory response is responsible for inflammatory mediators leak in the cardiovascular system, especially C-reactive protein (CRP),^[20,21]^ etc, which may be the risk factors for 28-day mortality. In fact, this has been confirmed by our this study.

Another evidence and explain of involving SAMI and high mortality is as follows. Previous study showed that the SOFA score was associated with MOF, while MOF with poor outcomes due to sepsis was also confirmed by previous studies,^[9,16,22]^ and most conditions included the cardiovascular failures.^[9,23]^ However, Our now study confirmed that the elevated initial MIMs score in non-survivors of SAMI was 6 times higher than in non-survivors. Also, we found the elevated MIMs score was more closely related to the mortality when compared with elevated SOFA scores and SIRS points. The elevated MIMs score was associated with SOFA score and mortality, which had lead to the poor outcome for patients with SAMI at 28 days follow-up. The mechanisms of MOF are related to cytokines and angiotensin conversion enzyme 2, both involved systemic inflammatory response/immune response.^[22–26]^ Moreover, the SIRS is involved massive cytokines released and lead to inflammation-derived injurious.^[23]^ Cytokine storm can cause devastating inflammatory conditions, which included MOF.^[25,26]^ These conditions can also be seen in correlation analysis between MIMs score and SOFA score/SIRS point.

Some limitations of this design must be addressed. First, this study was from 3 ICUs in a hospital sample. Moreover, those ICUs patients were from an emergency department of this hospital. Despite only a few patients were excluded either death or automatically out of the ICU within the first 24 hours. But we believe that maybe this population would have a little bias. Second, The morbidity and mortality of cardiac arrest was very high in global,^[27]^ and bacterial infection was a potential cause or complication of cardiac arrest.^[28]^ However, we excluded those patients who died within 24 hours in the ICU and they have been underwent a CPR. Most of those patients could have SAMI. Thus, this could overlook early mortality trends associated with SAMI. Third, we had little collected the data on those who have survived cardiac arrest. This may also produce bias in the incidence of SAMI. Fourth, the LOS was not related to outcome prediction. In fact, the highest MIMs score had a better prognostic value than the other MIMs scores derived variables. This may be because patients who present with a limited degree of myocardial injury and have a long ICU stay still have a high likelihood of survival. In addition, we did not collect septic shock data, we wonder if its MIMs scores will be higher.

## 5. Conclusions

The severity of SAMI was associated with elevated 4 MIMs scores, which was leading to high mortality during the ICU stay. It suggests that cognition this life-threatening SAMI is a major public health problem.

## Acknowledgments

We would like to thank everyone who contributed to the study, namely, the nurses and physicians who recorded the patient data and the related senior experts for reviewing the medical records in the ICU.

## Author contributions

**Conceptualization:** Ye-Ting Zhou, Dao-Ming Tong.

**Data curation:** Shao-Dan Wang, Dao-Ming Tong.

**Formal analysis:** Xin-Chun Gao, Shao-Dan Wang, Si-Wei Wang, Dao-Ming Tong.

**Funding acquisition:** Ye-Ting Zhou.

**Investigation:** Ye-Ting Zhou, Dao-Ming Tong.

**Validation:** Guang-Sheng Wang, Xin-Chun Gao, Si-Wei Wang.

**Visualization:** Guang-Sheng Wang, Xin-Chun Gao, Si-Wei Wang.

**Writing – original draft:** Dao-Ming Tong.

**Writing – review & editing:** Ye-Ting Zhou, Guang-Sheng Wang, Xin-Chun Gao, Shao-Dan Wang, Dao-Ming Tong.

## Supplementary Material


