# Supplementary material for: Sepsis-associated myocardial injury: Incidence and mortality
Source: Medicine (Baltimore). 2025 Jun 20;104(25):e42513. doi: 10.1097/MD.0000000000042513 (PMC12187275; doi:10.1097/MD.0000000000042513)
Supplement: Supplementary file 1 [file medi-104-e42513-s001.docx]

Supplemental Online Content

Supplementary Appendix A , Tables

Appendix Table 1. Condition of bacterial culture in sepsis patients (n=316)

|  | Total  (n=316) |
| --- | --- |
| Positive sputum culture,n(%)  Acinetobacter baumannii  Klebsiella pneumonae  E.coli  Staphylococcus aureus  Positive blood culture,n(%)  Gram negative bacilli  Acinetobacter baumannii  Klebsiella pneumonae  Pseudomonas aeuruginosa  E.coli  Heamephilus influenzae  Gram positive cocci  Staphylococcus aureus  Streplococcus pneumoniac  Enteroccus feecalis  Fungi  Positive urine culture,n(%) | 260(82.4)  113(35.9)  100(31.7)  63(20.0)  40(12.5)  92(29.0)  43 (46.7)  14(15.6)  11(12.2)  8(8.8)  7(7.8)  2(2.2)  51(53.3)  18(20.0)  16(17.7)  12(13.3)  2(2.2)  13(4.2) |
|  |  |

Appendix Table 2.The SAMI related subsequent multiple organ failureatthe 28days in the ICU

| Organ | Non-survivousl  (N=177) | Survivous  (N=139) | P value |
| --- | --- | --- | --- |
| Cardiovascular  Arrythmia/ atrial fibrillation,n(%)  Cardiac arrest/CPR,n(%)  Brain:delirium/coma,n(%)  Brain Death,n(%)  Lung: acute lung injury/ARDS,n(%)  Need mechanical ventilated,n(%)  Liver: elevatedbilirubin or liver enzymes,n(%)  Renal:elevated creatinine,n(%)  need kidney replacement therapy,n(%)  Coagulation: low platelets count,n(%)  VA-ECMO,n(%) | 34.2  96.1  98.3  3.9  90.7  99.4  36.7  53.8  35.3  18.6  No one | 21.7  19.9  66.8  0.0  59.3  76.7  29.4  47.9  18.1  10.1  No one | 0.017  0.000  0.000  0.021  0.000  0.000  0.183  0.495  0.010  0.055 |

Supplementary appendix B, Figures 1-3

Appendix Figure1


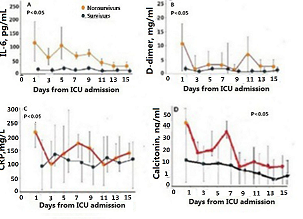




Appendix Figure1

Theseearly elevated levels of lL-6, D-dimer, CPR, and procalcitonin tended to downtrend gradually in the non-survivors,but there were still differences compared with survivors (all *P*<0.05)


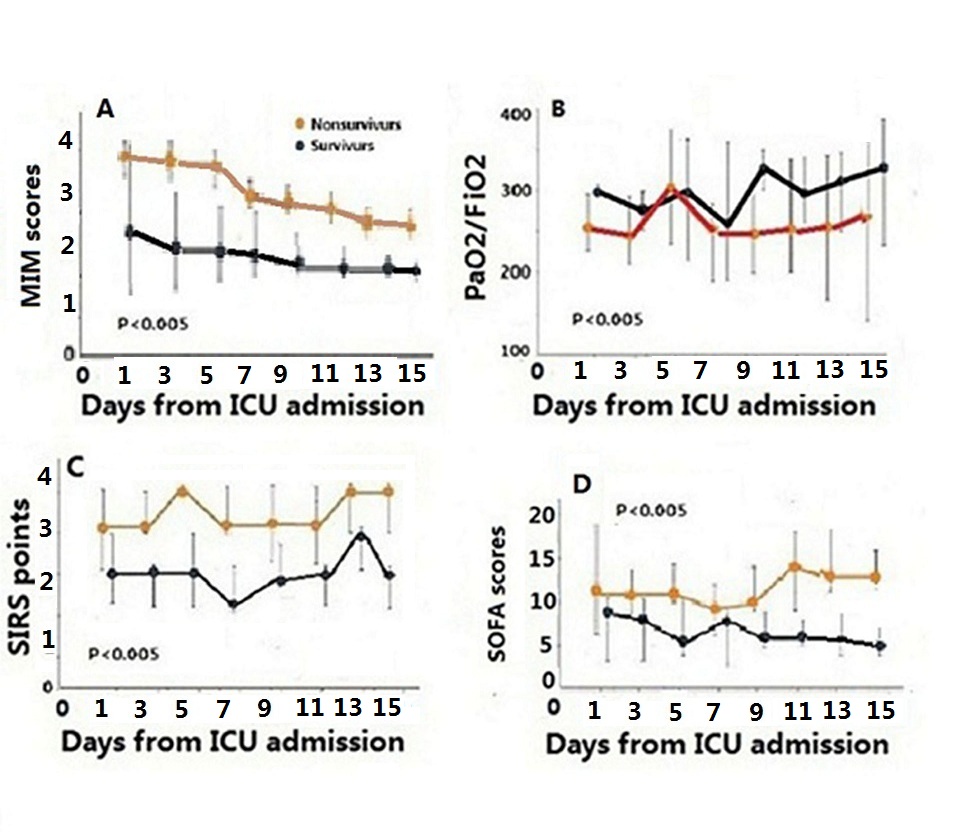


Appendix Figure 2.Changes in clinical parameters from SAMI during initial 15 days in ICU. Elevated MIM scores (A), lowered :PaO2/FiO2 (B), elevated SIRS points (C), and elevatedSOFA score (D) were notsignificant improved over time in non-survivors than in survivors(All p<0.005).


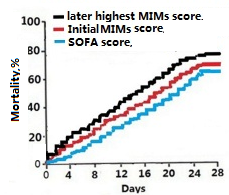


Appendix Figure 3. The correlation coefficient for mortality show tnatlater highest MIM score (r=0.75, 95%CI, 0.6609-0.8226,p<0.001) is higher than initial MIMs score(r=0.70,95%CI, 0.5764 -0.7729,p<0.001) and SOFA score (r=0.68, 95%CI,0.5959-0.7846,p<0.001) at 28 days in ICU.
